# Outcomes of implants placed after osteotome sinus floor elevation without bone grafts: a systematic review and meta-analysis of single-arm studies

**DOI:** 10.1186/s40729-021-00358-3

**Published:** 2021-08-09

**Authors:** Mingfu Ye, Wenjun Liu, Shaolong Cheng, Lihui Yan

**Affiliations:** Department of Oral Implantology, Stomatological Hospital of Xiamen Medical College, Xiamen Key Laboratory of Stomatological Disease Diagnosis and Treatment, 1309, Lvling Road, Xiamen, 361008 Fujian Province People’s Republic of China

## Abstract

**Background:**

The aim of this study is to evaluate the implant survival/success rate, gain in alveolar bone height, crestal bone loss, and complications associated with implants placed in the posterior maxilla after osteotome sinus floor elevation without bone substitutes.

**Methods:**

The electronic databases, such as MEDLINE, EMBASE, CENTRAL, and SCOPUS were systematically and manually searched for publications in peer-reviewed journals. The included articles were subjected to qualitative and quantitative analyses, and the meta-analysis was carried out for single-arm studies. Methodological quality assessment was made for all the included studies.

**Results:**

The included studies were of moderate quality, with the overall implant success and survival rates of 98.3% and 97.9% respectively. The most frequent intra-surgical complication was sinus membrane perforation, accounting for 3.08% of the total implants with reported perforations. The overall crestal bone loss in patients with immediate implants placed with OSFE after a 5-year follow-up was 0.957 mm 95%CI (0.538, 1.377).

**Conclusion:**

Within the limitations of this review, it can be concluded that the survival and success rates of implants placed immediately along with OSFE without any bone substitutes are acceptable and show adequate implant stability with less crestal bone loss over 5 years.

## Introduction

Dental implants provide a strong foundation for fixed (permanent) or removable replacement teeth that are essential for the improvement of appearance, speech, eating, comfort, self-esteem, and oral health of the patients [1]. A loss of the natural dentition leads to a reduction of occlusal forces that activate a series of bone remodeling processes in the alveolar bone, causing pressure-threshold-regulated bone atrophy [1]. However, there is still not enough scientific evidence to determine whether osteoclastic bone resorption is pressure-threshold-regulated or proportionally pressure-dependent. Moreover, after tooth extraction, there is an increase in the osteoclastic activity of the periosteum of the maxillary sinus floor, leading to sinus maxillary sinus pneumatization and expansion into the alveolar bone crest [2]. Maxillary sinus pneumatization is a serious obstacle to oral implantology [2]. Therefore, there is a great need for specific surgical procedures to partially or totally reduce the expanded volume of this cavity. Several grafting techniques based on using autogenous bone (either alone, mixed with a bone-substituting biomaterial, or biomaterial only) are now available. Insufficient alveolar bone height, width, and density, as well as quality and quantity of posterior edentulous maxillary bone, are common limiting factors for placement of dental implants in the posterior maxillary region. These factors can increase incidences of implant failure and complications and worsen overall clinical outcomes of dental implant treatments [3]. Surgical sinus floor elevation (SFE) can significantly increase the height of bone available for implant placement. For dental implant placement, two main sinus floor elevation approaches can be used—direct and indirect. Direct SFE is a lateral window sinus grafting approach that is used for treating cases with a residual bone height of less than 5.0 mm. This approach allows to increase bone height to > 5.0 mm but usually requires a 6–9-month delay in subsequent implant placement. Indirect SFE is a transalveolar approach that condenses bone grafting materials under the Schneiderian membrane in the presence of at least 5 mm of residual bone. This approach allows gaining approximately 3–5.0 mm of bone height within the sinus with a simultaneous implant placement [4].

The use of bone grafts for sinus augmentation, irrespective of the technique used, has been associated with a high success rate despite certain shortcomings, such as a need for a second surgical site for autogenous bone harvesting, increased rate of complications, higher cost, and increased surgical time. Lundgren et al. described spontaneous bone formation below the sinus floor after cyst enucleation, suggesting that proliferative and regenerative proprieties of the sinus membrane may have a potential for bone formation [5]. This concept led to a number of studies in which successful implant placement and rehabilitation were carried out without using bone grafts. These studies have demonstrated a guided tissue regeneration process, where bone deposition and new bone formation are induced by the blood clot in the void that is created after sinus augmentation [6].

In 2019, Rawat et al. conducted a prospective controlled clinical trial of 21 patients with 26 implants by indirect sinus lift with simultaneous implant placement without bone graft. This study demonstrated a predictable successful osseointegration with osteotome sinus floor elevation without bone graft, and spontaneous new bone formation [4]. A prospective study by Merheb et al. [7] compared the 5-year progression of implant stability in grafted and non-grafted sites in 12 patients with ≤ 4-mm initial bone height in the posterior maxilla. The implants were positioned using osteotome sinus floor elevation. This study showed that the stability of implants positioned with osteotome sinus floor elevation in non-grafted sites is similar to that of implants placed in grafted sites. A randomized controlled trial by Qian et al. [8] evaluated long-term clinical and radiographic outcomes of implants placed using osteotome sinus floor elevation (OSFE) with or without bone grafting in 45 patients with 4.58 ± 1.28 mm of average residual bone height. The study concluded that OSFE with or without grafting gives similar clinical outcomes with comparable alveolar bone gain. Since then several new studies have been published. The aim of the current study is to provide updated pooled evidence and meta-analysis by systematically searching the literature for all single-arm studies that evaluate the outcomes of implants placed in posterior maxillae after osteotome sinus floor elevation without bone substitutes.

## Methods

### Review methodology

This systematic review and meta-analysis of single-arm studies was carried out in strict accordance with Preferred Reporting Items for Systematic Review and Meta-Analyses (PRISMA) guidelines [9]. The protocol for smooth conduction of the systematic review was prepared a priori.

### Review question

What is the survival/success rate of the implants placed in the posterior maxilla after osteotome sinus floor elevation without any bone substitutes?

What is the gain in alveolar bone height, crestal bone loss?

What intra-surgical and post-surgical complications were reported with the implants placed in the posterior maxilla after osteotome sinus floor elevation without any bone substitutes?

### Designing PICO

The description of PICO is as follows:

**Table:** 

*Population/type of participants*	The patients indicated for immediate dental implant placement in posterior maxillae with insufficient residual bone height requiring sinus elevation
*Type of intervention*	Immediate dental implant placement following osteotome sinus floor elevation without any additional bone substitutes
*Comparison*	Not applicable (single-arm studies)
*Outcomes*	Survival rate, success rate, gain in alveolar bone height, crestal bone loss around implants, intra-surgical and post-surgical complications

### Search strategy

A comprehensive search was carried out in 4 electronic databases, MEDLINE, EMBASE, CENTRAL, and SCOPUS, using a series of relevant keywords: *Maxillary sinus*, *Dental Implant*, *Sinus augmentation*, *Sinus elevation*, *Crestal sinus elevation*, *Summer’s osteotome*, *Osteotome sinus floor elevation*, *OSFE*, *Indirect sinus lift*, *Immediate Implant*, *Survival rate*. We searched each database from 1979 up to 10th February 2021. A manual search was also carried out in peer-reviewed international indexed journals, such as Clinical Implant Dentistry and Related Research, Clinical Oral Implant Research, Implant Dentistry, International Journal of Oral and Maxillofacial Implants, Journal of Clinical Periodontology, Journal of Periodontal and Implant Science, Journal of Periodontology, and Quintessence International, from inception till January 2021. The bibliographies of previously conducted relevant systematic reviews or randomized clinical trials were additionally screened for any potentially eligible articles. The search was limited to the studies published in the English language only.

Articles retrieved from the digitalized and manual sources were imported into a citation manager software to remove the duplicates, and the final set of retrieved studies was screened by looking at titles and abstracts on the basis of relevancy. The potentially eligible articles were then subjected to full text analysis.

### Selection of studies

The study selection was carried out by two independent reviewers.

The inclusion criteria were as follows:

- Articles published in the English language
- Single-arm clinical studies with human subjects
- Articles employing OSFE alone without any bone substitute along with simultaneous placement of dental implant
- Articles with RBH measurements
- Articles with a minimum sample size of 10 and a minimum follow-up of 6 months–1 year
- Articles reporting implant survival/success rate, alveolar bone gain, crestal bone loss, or post-surgical adverse events

The articles not reporting the outcomes, or multiple publications with the same cohort, or employing ridge split or any additional augmentation procedures, were excluded.

### Data selection and extraction

Data from the included articles were collected by two independent reviewers, and the information was entered into the excel sheet under the following domains: study design, sample size, gender, age range; smokers; number and location of implants placed; make, diameter, and length of implants placed; osteotome technique; follow-up months; etc. The primary outcomes assessed were implant survival, implant success, gain in alveolar bone height, and mean crestal bone loss around the implants placed. Secondary outcomes included the intra-surgical and post-surgical complications observed across the included studies. The authors were contacted through email for clarification and in case of any missing relevant information.

### Data synthesis

The retrieved data was subjected to both qualitative and quantitative synthesis. Demographic and interventional characteristics were included in the table and summarized. In the case of two or more studies assessing similar outcomes, the quantitative items were subjected to single-arm pooled meta-analysis using the Open Meta-analyst 2.0 software. The pooled estimate of gain in alveolar bone height and mean crestal bone loss was expressed as mean and standard deviation with 95% confidence interval (CI). The dichotomous data pertaining to implant success/survival was expressed as pooled odd’s ratio (OR) with 95% CI. The heterogeneity among the included studies was assessed using i^2^ statistics. The i^2^ value greater than 70% was considered high heterogeneity, and less than 40% was considered low heterogeneity.

### Quality assessment

The quality assessment of the included studies was carried out using the methodology assessment criteria adopted by Clementini et al., by judging the following domains: appropriateness in statistical analysis, validated measurements, reports of loss to follow-up, defined inclusion and exclusion criteria, and proper sample selection.

## Results

**Fig. 1:**
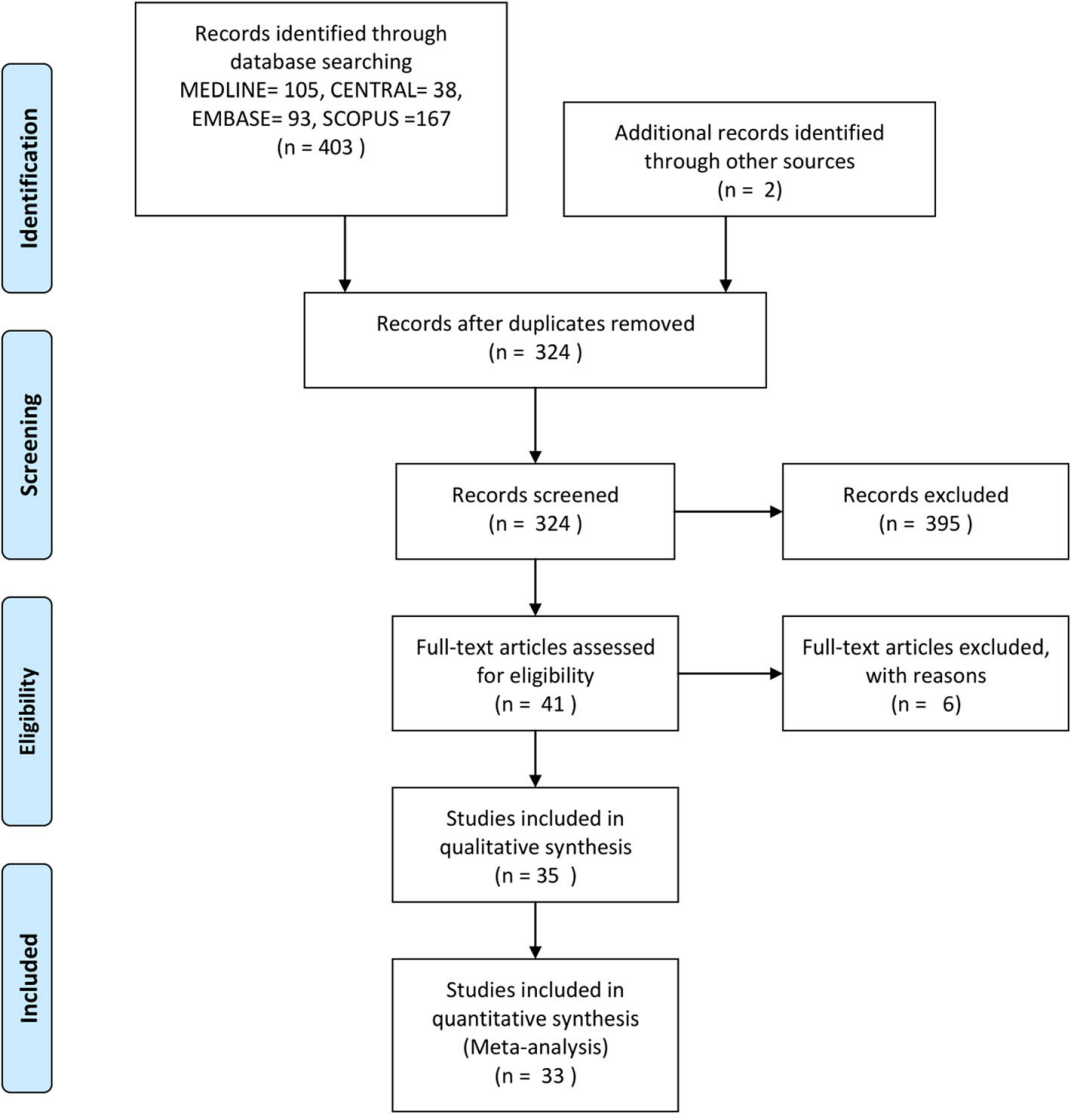
Study selection process (PRISMA)

A pool of 324 articles were retrieved from digitalized and manual searches and screened based on titles and abstracts. A total of 41 potentially eligible articles were selected for full text assessment. After evaluating inclusion and exclusion criteria, 35 articles [4, 7, 8, 10–41] were included, and 6 manuscripts [42–47] were excluded. The detailed study selection process is summarized in Fig. 1.

Seven clinical trials [4, 8, 10, 16, 18, 24, 33], fifteen prospective clinical studies [7, 13–15, 17, 21, 22, 26–29, 31, 32, 37, 39], twelve retrospective cohort studies [11, 12, 19, 20, 23, 25, 30, 34–36, 40, 41], and 1 case series [27] were included in this systematic review. The age of the patients included in the studies ranged from 17 to 90 years. Eight studies [8, 10, 18, 26, 29, 34, 36, 39] reported the inclusion of smokers, and two studies reported exclusion of smokers [13, 15]. The rest of the studies did not report the smoking status of the patients. The follow-up period ranged between 6 months and 16 years. Overall, data on 2267 patients with a total of 3390 dental implants were reported in the 35 selected studies. The pre-operative residual bone height (RBH) ranged from 2 to 13.5 mm. The diameters of the implants varied between 3.3 and 7.0 mm, and the length of the implants ranged from 6 to 15 mm. The highest reported success rate was 100%, and the lowest was 95%. Demographic and interventional characteristics of the included studies are summarized in Table 1 and Table 2, respectively.

**Table 1:** Demographic characteristics of included studies

S.L.no.	Author	Year	Country	Centers	Surgeons	Study design	Sample size	Gender	Age range	Smokers
1	Leblebicioglu et al. [10]	2005	Turkey	1	NR	Randomized controlled clinical trial	40	21F, 19M	46.7 years	Yes
2	Jurisic et al. [11]	2008	Serbia	1	2	Retrospective cohort	33	26F, 35M	38–64 years	NR
3	Schmidlin et al. [12]	2008	Switzerland	1	2	Retrospective cohort	24	15F, 9M	61.9 ± 10.3 years	NR
4	Gabbert et al. [13]	2009	Germany	1	2	Prospective clinical study	36	20F, 16F	20–76 years	No
5	Nedir et al. [14]	2009	Switzerland	1	2	Prospective clinical study	32	NR	39–82 years	NR
6	Pjetursson et al. [15]	2009	Switzerland	1	NR	Prospective clinical study	181	NR	17–90 years	No
7	Lai et al. [16]	2010	Switzerland	1	NR	Clinical trial	202	NR	20–68 years	NR
8	Nedir et al. [17]	2010	Switzerland	1	NR	Prospective clinical study	17	14F, 3M	38–69 years	NR
9	Fornell et al. [21]	2011	Sweden	1	NR	Prospective clinical study	14	7M, 7F	34–75 years	NR
10	He et al. [23]	2011	China	NR	NR	Retrospective cohort	22	10F, 12M	19–70 years	NR
11	Senyilmaz et al. [18]	2011	Turkey	NR	NR	Pilot study	17	9F, 8M	55 years	Yes
12	Volpe et al. [25]	2011	Sweden	NR	NR	Retrospective cohort	20	15F, 5M	48 years	NR
13	Zahran et al. [22]	2011	Egypt	NR	NR	Prospective clinical study	64	34F, 30M	35–72 years	NR
14	Bruschi et al. [19]	2012	Italy	1	1	Retrospective cohort	46	29F, 17M	26–83 years	NR
15	Fermergard et al. [20]	2012	Sweden	NR	NR	Retrospective cohort	36	NR	64 ± 12 years	NR
16	Si et al. [24]	2013	China	1	NR	Randomized controlled clinical trial	20	NR	≥ 18 years	NR
17	Brizuela et al. [26]	2014	Spain	1	1	Prospective clinical trial	37	22F, 15M	31–68 years	Yes
18	Gu et al. [31]	2016	China	1	NR	Prospective clinical study	28	13F, 15M	19–78 years	NR
19	Nedir et al. [27]	2014	Switzerland	1	NR	Case series	7	NR	47.5 ± 18.4 years	NR
20	Bassi et al. [28]	2015	Sweden	1	NR	Prospective clinical study	17	NR	NR	NR
21	Markovic et al. [32]	2015	Serbia	2	NR	Prospective clinical trial	45	NR	18–56.7 years	NR
22	Nedir et al. [33]	2016	Switzerland	1	NR	Prospective clinical study	17	14F, 3M	38–69 years	NR
23	Spinelli et al. [29]	2015	Switzerland	1	NR	Prospective clinical study	39	17F, 12M	33–76 years	Yes
24	French et al. [30]	2016	Canada	NR	NR	Retrospective cohort	541	279F, 262M	18–88 years	NR
25	Nedir et al. [38]	2017	Switzerland	1	NR	Randomized controlled clinical trial	9	NR	57.6 ± 4.7 years	NR
26	Si et al. [34]	2016	China	1	NR	Retrospective cohort	80	37F, 43M	25–70 years	Yes
27	Zill et al. [35]	2016	Germany	1	NR	Retrospective cohort study	113	NR	31–84 years	NR
28	Caban et al. [36]	2017	Sweden	1	1	Retrospective cohort	25	11F, 14M	44–84 years	Yes
29	Cheng et al. [37]	2017	China	NR	NR	Prospective clinical study	29	13F, 35M	43–71 years	NR
30	Abi Najm et al. [39]	2018	Switzerland	NR	NR	Prospective clinical study	17	14M, 3F	38–69 years	Yes
31	Yang J et al. [40]	2018	China	1	1	Retrospective cohort	40	19F, 21M	22–70 years	NR
32	Merheb et al. [7]	2019	Switzerland	1	NR	Prospective clinical study	12	9F, 3M	57.6 ± 4.7 years	NR
33	Qian et al. [8]	2020	China	1	NR	Randomized controlled clinical trial	22	NR	≥ 18 years	Yes
34	Rawat et al. [4]	2019	India	NR	NR	Randomized controlled clinical trial	21	NR	NR	NR
35	Nahlieli et al. [41]	2019	Turkey	NR	NR	Retrospective study	331	NR	NR	NR

*NR* not reported, *M* male, *F* female

**Table 2:** Interventional characteristics of the included studies

S.L. no.	Author	Year	Location	No. implants	Make of implant	Diameter of implant placed	Length of implant placed	Healing time	Bone quality	Follow-up
1	Leblebicioglu et al. [10]	2005	First premolar (16%) second premolar (26%), first molar (52%), second molars (6%)	75	NR	NR	8 mm	6 months	D3, D4	25 months
2	Jurisic et al. [11]	2008	Premolar (NR), molar (NR)	40	Straumann with SLA	4.03 + 0.13	10.72 + 0.76	NR	NR	3 years
3	Schmidlin et al. [12]	2008	Premolar (10), molar (14)	24	NR	4.4 + 0.4	8.6 + 1.3	NR	NR	17.4 + 18.4 months
4	Gabbert et al. [13]	2009	Premolar (41), molar (51)	92	ITI solid screw and Nobel Biocare	NR	8 mm, 10 mm, 11.5 mm, 12 mm		NR	1.2 + 0.69 years
5	Nedir et al. [14]	2009	Premolar (17), molar (37)	54	Straumann	4.8 mm, 6.5 mm	8 mm, 10 mm	4.2 ± 2.6 months	D1, D2, D3, D4	1 year
6	Pjetursson et al. [15]	2009	Second premolar (46%), first molar (35%), first premolar (14%), second molar and canine (5%)	252	Straumann	4.1 mm, 4.8 mm, 3.3 mm	6 mm, 8 mm, 10 mm, 12 mm	4–6 months	D4	3.2 years
7	Lai et al. [16]	2010	NR	191	Straumann	4.1 mm, 4.8 mm	6 mm, 8 mm, 10 mm, 12 mm	NR	NR	3 & 6 months
8	Nedir et al. [17]	2010	Premolar (9), molar (16)	25	Straumann	NR	6 mm, 8 mm, 10 mm	3–4 months	D3, D4	3 & 5 years
9	Fornell et al. [21]	2011	NR	21	SLActive	4.1 mm, 4.8mm	10 mm	NR	NR	1 year
10	He et al. [23]	2011	Premolar (3), molar (24)	27	BEGO	4.7 ± 0.4 mm	10 ± 1.0 mm	NR	D1, D2, D3, D4	2 years
11	Senyilmaz et al. [18]	2011	Premolar (4), molar (23)	27	Straumann	4.1 mm	8 mm, 10 mm	8–12 weeks	NR	2 years
12	Volpe et al. [25]	2011	Premolar (19), molar (10)	29	NR	4 mm	NR	6 months	NR	16.4 months
13	Zahran et al. [22]	2011	NR	108	OsteoCare™ Maxi-Z Flat-End	3.75 mm, 4.5 mm	8 mm, 10 mm,12 mm	6 months	D4	1 year
14	Bruschi et al. [19]	2012	NR	66	Frialit, PILOT	4.5 mm,5.5 mm,6.5 mm,4.7 mm, 5.7 m, 6.7 mm	13 mm, 15 mm	NR	NR	1, 5, 10, & 16 years
15	Fermergard et al. [20]	2012	NR	53	Astra Tech	4.5 mm	9 mm,11 mm,13 mm	NR	NR	1 & 3 years
16	Si et al. [34]	2016	Premolar (9), molar (11)	20	SLA	4.1 mm, 4.8 mm	6 mm, 8 mm, 10 mm	NR	NR	6,12,24,36 months
17	Brizuela et al. [26]	2014	Premolar (13), molar (23)	36	Klockner	3 mm, 4.1 mm, 5 mm	8 mm, 10 mm	NR	NR	2 years
18	Gu et al. [31]	2016	NR	41	SLA	NR	NR	NR	NR	1, 3, & 5 years
19	Nedir et al. [27]	2014	First molar	7	SLA	4.1 mm, 4.8 mm	8 mm, 10 mm	12 weeks	NR	1, 3, 5, & 10 years
20	Bassi et al. [28]	2015	NR	25	NR	4.3 mm	13 mm	NR	NR	3 & 51 months
21	Markovic et al. [32]	2015			SLActive-BL	4.1 mm	10 mm	6 months		1 & 2 years
22	Nedir et al. [33]	2016	Premolar (9), molar (16)	25	SLA	4.8 mm	6 mm, 8 mm, 10 mm	3.1 ± 0.4 mm	NR	1, 3, 5, & 10 years
23	Spinelli et al. [29]	2015	Premolar (NR), molar (NR)	66	NobelSpeedy Groovy and NobelActiveInternal, Nobel Biocare AB	4 mm, 4.8 mm	10 mm, 11.5 mm, 13 mm	5 months	NR	3 years
24	French et al. [30]	2016	NR	926	Straumann, Nobel Biocare	4.1 mm 4.3 mm, 4.8 mm, 5mm	6 mm, 8 mm, 10 mm, 12 mm, 13 mm	NR	NR	10 years
25	Nedir et al. [38]	2017	Premolar (NR), molar (NR)	17	TE SLActive	4.1 mm, 4.8mm	8 mm	2.6 ± 0.9mm	NR	1,3, & 5 years
26	Si et al. [34]	2016	Premolar (15), molar (81)	96	Straumann	4.1 mm, 4.8 mm	8 mm, 10 mm, 12 mm	NR	NR	4, 5, 6, 7, 8, & 9 years
27	Zill et al. [35]	2016	Premolar (66), Molar (167)	233	Straumann solid screw transmucosal implants	3.3 mm, 4.1 mm, 4.8 mm	6 mm, 8 mm, 10 mm,12 mm	3 months	NR	5 years
28	Caban et al. [36]	2017	First premolar (12), second premolar (18), First molar (4)	34	Astra Tech	4.5 mm	9 mm, 11 mm, 13 mm	3.5 months	NR	10 years
29	Cheng et al. [37]	2017	Second premolar (6), first molar (28), second molar (14)	48	Bicon, Nobel Replace	4.9 mm	6.8 mm	3–6 months	NR	6 months
30	Abi Najm et al. [39]	2018	First premolar (2), second premolar(8), first molar (10), second molar (1)	21	Straumann	NR	6 mm, 8 mm, 10 mm	NR	NR	10 years
31	Yang J et al. [40]	2018	NR	27	Bicon	4.5 mm, 5 mm	6 mm, 8 mm	6 months	NR	18 months
32	Merheb et al. [7]	2020	NR	20	TE SLActive	4.1 mm, 4.8 mm	8 mm	8 weeks	D2, D3, D4	5 years
33	Qian et al. [8]	2020	NR	22	Straumann with SLA	4.1 mm, 4.8 mm	6 mm, 8 mm, 10 mm	NR	NR	1, 3, 5, & 10 years
34	Rawat et al. [4]	2019	Second premolar (26%), first molar (40%), second molar (33%)	26	Pitt Easy Puretex	3.25 mm, 4 mm, 4.9 mm	10 mm, 12 mm	6 months	NR	3 & 6 months
35	Nahlieli et al. [41]	2019	NR	722	NR	3.75 mm, 4.20 mm	11.5 mm, 13 mm	6 months	NR	6 months–7 years

*NR* not reported

Intraoperative membrane perforation was the most frequently observed intraoperative complication and was reported by 22 studies [7, 10–17, 22, 24, 26–31, 33–35, 37, 39]. Out of 22 studies, 11 studies [7, 17, 22, 24, 26–29, 34, 35, 37] did not report any tear or perforation in the sinus membrane. Membrane perforation occurred in 88 cases out of 2858 implants placed, accounting for 3.08% of the total implants with reported perforations. Postoperative nosebleed, paroxysmal vertigo, and infections were observed in few studies, however, they were less frequent. The details regarding the intra-surgical and post-surgical complications are provided in Table 3.

**Table 3:** Adverse events reported among the included studies

S.L. no.	Author	Year	Sample size	No. of implants	No. (%) of membrane perforations	Postoperative nosebleed	Postoperative paroxysmal vertigo	Postoperative infection
1	Leblebicioglu et al. [10]	2005	40	75	2 (3.70)	0	N/A	0
2	Jurisic et al. [11]	2008	33	40	7	N/A	N/A	3
3	Schmidlin et al. [12]	2008	24	24	2 (8.33)	1	0	N/A
4	Gabbert et al. [13]	2009	36	92	24 (26)	N/A	N/A	0
5	Nedir et al. [14]	2009	32	54	5 (9.25)	0	N/A	0
6	Pjetursson et al. [15]	2009	181	252	26 (10.40)	N/A	9	0
7	Lai et al. [16]	2010	202	280	12 (4.29)	3	0	2
8	Nedir et al. [17]	2010	17	25	0	N/A	N/A	N/A
9	Fornell et al. [21]	2012	14	21	N/A	N/A	N/A	N/A
10	He et al. [23]	2013	22	27	N/A	N/A	N/A	N/A
11	Senyilmaz et al. [18]	2011	17	27	N/A	N/A	N/A	N/A
12	Volpe et al. [25]	2013	20	29	N/A	N/A	N/A	N/A
13	Zahran et al. [22]	2012	64	108	0	0	N/A	0
14	Bruschi et al. [19]	2012	46	66	N/A	4	N/A	N/A
15	Fermergard et al. [20]	2012	36	53	N/A	N/A	N/A	N/A
16	Si et al. [24]	2013	20	20	0	0	N/A	0
17	Brizuela et al. [26]	2014	37	36	0	0	N/A	0
18	Gu et al. [31]	2016	28	41	2	0	N/A	0
19	Nedir et al. [27]	2014	7	7	0	N/A	N/A	N/A
20	Bassi et al. [28]	2015	17	25	0	0	O	0
21	Nedir et al. [33]	2016	17	25	4 (16)	1	N/A	0
22	Spinelli et al. [29]	2015	39	66	0	0	0	0
23	French et al. [30]	2016	541	926	1	N/A	0	1 (0.1%)
24	Nedir et al. [38]	2017	9	17	N/A	N/A	0	1
25	Si et al. [34]	2016	80	96	0	0	N/A	0
26	Zill et al. [35]	2016	113	233	0	N/A	N/A	N/A
27	Caban et al. [36]	2017	25	34	N/A	N/A	N/A	0
28	Cheng et al. [37]	2017	29	48	0	0	N/A	0
29	Abi Najm et al. [39]	2018	17	21	3	N/A	N/A	1
30	Yang J et al. [40]	2018	40	27	N/A	N/A	N/A	N/A
31	Merheb et al. [7]	2020	12	20	0	N/A	N/A	0
32	Qian et al. [8]	2020	22	22	N/A	N/A	N/A	0
33	Rawat et al. [4]	2019	21	26	N/A	N/A	N/A	N/A

*N/A* data not available

### Meta-analysis

Fourteen different brands of implants were used; 5 articles [10, 12, 25, 28, 41] did not report any information on the dental implant brands; 4 studies [10, 13, 17, 31] did not provide any information on the dental implant diameters.

The quantitative data retrieved from the parameters assessed in five included studies [10, 14, 18, 22, 32] were pooled and the overall estimate with 95% CI was obtained. Most of the studies used success criteria described by Buser et al. [48] and Albrektsson et al. [49].

**Fig. 2:**
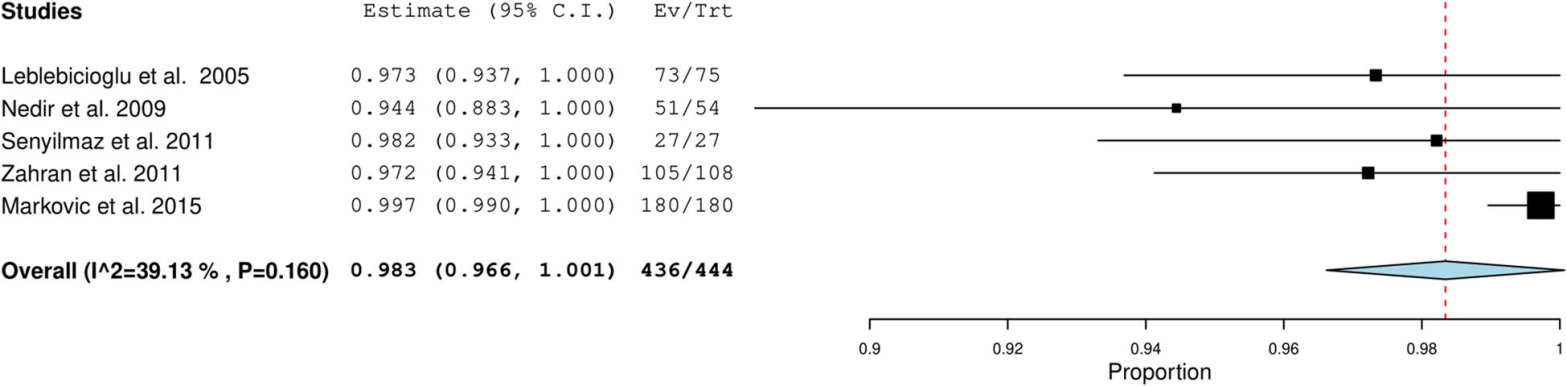
Pooled estimate of the implant success rate among the included studies

**Fig. 3:**
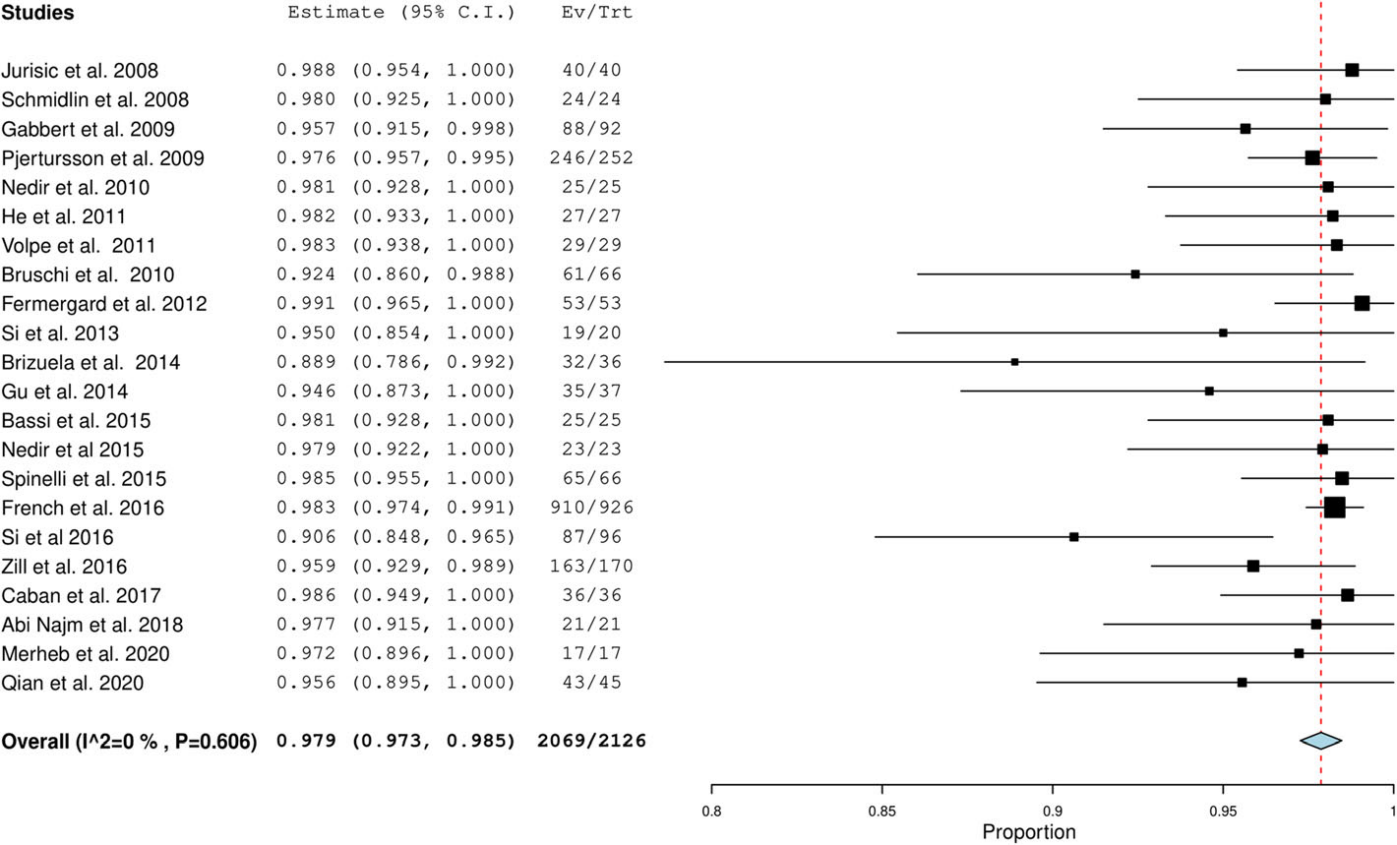
Pooled estimate of the implant survival rate among the included studies

The overall implant success rate was 98.3 (96.6–100) % (Fig. 2) with low heterogeneity (39.13%). Pooled survival rate of the twenty-two included studies [7, 8, 11–13, 15, 17, 19, 20, 23–26, 28–31, 33–36, 39] was 97.9% (97.3, 98.5) with 0% heterogeneity (Fig. 3).

**Fig. 4:**
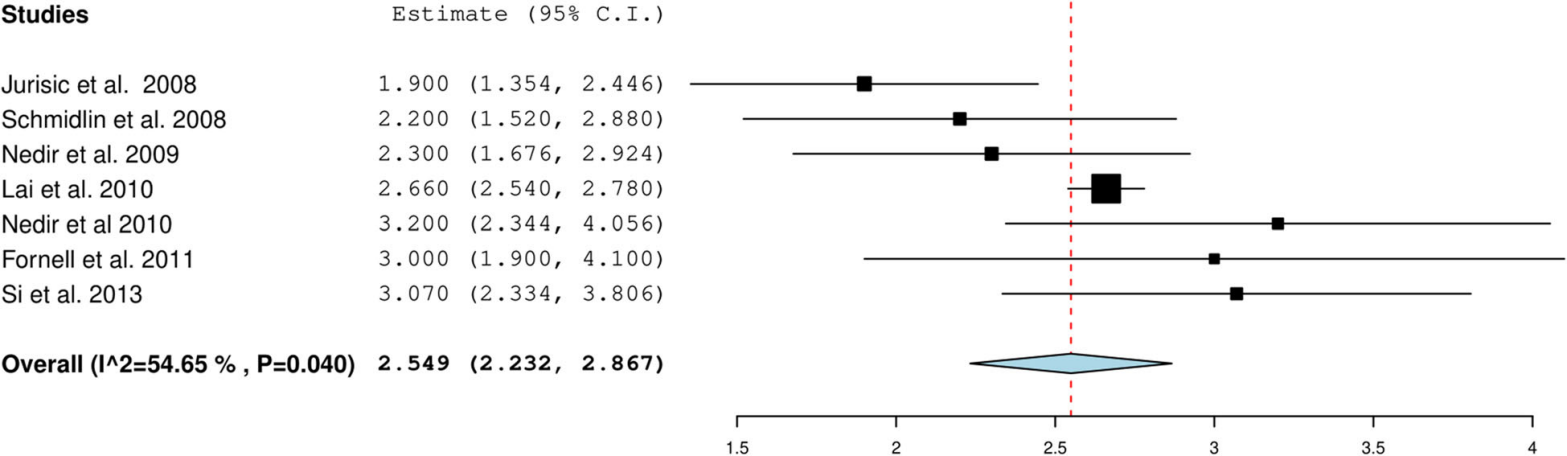
Pooled estimate of gain in alveolar bone height with RBH less than 6mm

**Fig. 5:**
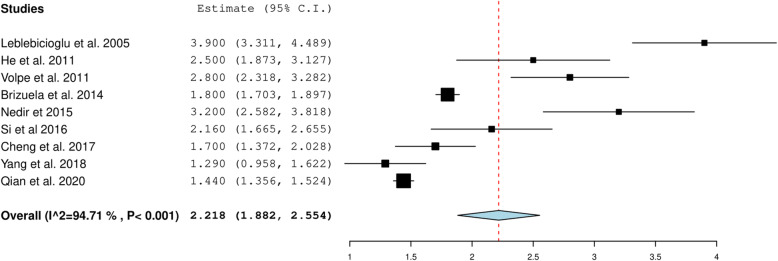
Pooled estimate of gain in alveolar bone height with RBH more than 6mm

**Fig. 6:**
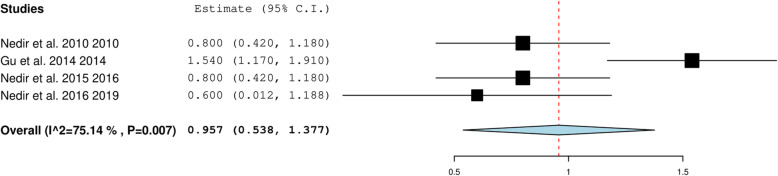
Pooled estimate of crestal bone loss in immediate implants placed with OSFE after a 5-year follow-up

The overall gain in the alveolar bone height was 2.459 mm 95%CI (2.232, 2.867) when the included studies describing < 6-mm RBH were pooled (Fig. 4). For studies with > 6-mm RBH, the overall gain was 2.218 mm, 95% CI (1.882, 2.554) (Fig. 5). The heterogeneity between the studies was high (94.71%), possibly due to the variation in length of implants that ranged from 6 to 15 mm and the variability in the pre-operative RBH. The overall crestal bone loss in immediate implants placed with OSFE after a 5-year follow-up was 0.957 mm, 95%CI (0.538, 1.377) (Fig. 6).

**Fig. 7:**
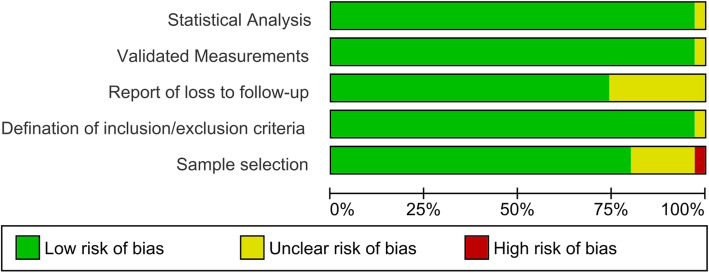
Methodological quality assessment of included studies

The quality of the included studies was moderate. One of the included studies [27] was a case series study, with a high risk in sample selection. However, most of the studies were ranked at low to moderate risk for appropriateness in statistical analysis, validated measurements, report of loss to follow-up, defined inclusion and exclusion criteria, and proper sample selection. The methodological quality assessment summary of included studies is provided in Fig. 7.

## Discussion

This systematic review and meta-analysis included 35 studies with a total of 3390 dental implants in 2267 patients.

The included studies reported both implant success and implant survival rates. The implant success rate is determined according to predefined success criteria [50]. The included studies reporting success rates employed one of the two success criteria described by Alberktson et al. [49], and Buser et al. [48], respectively. One included study [15] used different success criteria based on the clinical and radiological parameters such as distance between implant shoulder and mucosal margin, probing pocket depth, attachment level, and marginal bone level. The study was therefore not included in the pooled estimation of implant success rate. The overall implant success was estimated in only five out of 35 studies, showing a rate of 98.3%. The implant survival rate refers to the number of implants remaining in the patient’s mouth until the end of the follow-up period. The overall estimate of implant survival in our study was 97.9 %.

The implant success/survival can be influenced by numerous factors, implant dimension, surface characteristics, host factors, surgical technique, or any postoperative complications or infections [51]. The implant length reported in the included articles ranged from 6, 8, 10, 11.5, 13, and 15 mm. Majority of included studies reported length between 8 and 13 mm. Only 9 studies [8, 15–17, 30, 33–35, 39] used 6-mm length implants. One of the included articles [15] assessed the success rate relative to the length of the implant placed. According to Pjetursson et al. [15], the success rate of 6-mm trans-alveolar short implants placed with OSFE was 47.6%, while 8, 10, and 12-mm implants had success rates of 88.7%, 88.8%, and 100% respectively. The use of short implants resulted in reduced success/survival rate over a period of time. However, at the same time, it could reduce the chances of membrane tears.

The most common and frequent diameter of implants among the included studies ranged between 4 and 5 mm. However, only one study assessed implant survival in relation to the different implant diameters [16]. Lai et al. [16] showed that 161 implants with a diameter of 4.1 mm had a 95.15% survival rate, while 115 implants with a diameter of 4.8 mm had a survival rate of 96.62% [16].

Implant type as well as its surface characterization could also affect the implant success/survival rate. Sand-blasted, large-grit, acid-etched threaded implants were one of the most common types of implants used in the included articles. The SLA-treated surface results in increased bone-to-implant contact due to the elevated level of osteoblast proliferation and cellular adhesion at the surface of the dental implant [52]. These factors play a significant role in the process of osseointegration and aid in improving the wettability of the implant which is essential for better osseointegration in closed spaces like sinuses filled with blood clots.

The most frequent intra-surgical complication reported in the included studies was sinus membrane perforation, which occurred in 88 cases out of 2858 implants placed, 3.08% of the total implants with reported perforations. These results are in agreement with a previous systematic review by Tan et al. [53] that reported a total of 3.8% of perforations among 1776 implants assessed. A study by Del Fabbro et al. [54] also revealed 4.2% perforations out of a total of 3131 implants.

The endo-sinus bone gain is relative to the length of the implant [55]. Our analysis showed that the overall gain in the alveolar bone height was relatively higher in studies with < 6-mm RBH than in studies with > 6-mm RBH (2.459 mm 95%CI (2.232, 2.867) as compared to 2.218 mm 95% CI (1.882, 2.554)). The heterogeneity among the included studies was high, probably due to possible confounding factors, such as the different lengths of the dental implants, RBH ranging from 2.1 to 6 mm, and inclusion of smokers among the participants. Smoking could be a detrimental factor leading to implant failure. A study by Barone et al. [56] concluded that the postoperative infection rate was higher in smokers compared to non-smokers. This was further supported by the observation by Cha et al. [57] that smoking could be a possible factor of implant failure in immediate implants placed after OSFE. In the present systematic review, the included studies were heterogeneous, and the effect of smoking on any of the parameters could not be assessed.

A prospective randomized controlled trial by Nedir et al. 2017 [38] showed that the mean crestal bone loss at the end of 5 years was 0.6 + 1.1 mm. The overall crestal bone loss in immediate implants placed with OSFE after a 5-year follow-up was 0.957 mm 95%CI (0.538, 1.377). The crestal bone loss around implants is observed at a higher rate in the first year of functional loading. After that, the marginal bone remains relatively stable in well-placed, properly osseo-integrated implants.

## Conclusion

Within the limitations of this review, it can be concluded that the survival and success rates of implants placed immediately along with OSFE without any bone substitutes are 97.9 and 98.3 %, respectively. The most common complication observed with this technique was membrane perforation (up to 3.07% of the cases) that did not affect the survival of implants. OSFE showed improved alveolar bone height in the posterior maxilla with RBH < 6 mm and relatively stable crestal bone loss at the end of a 5-year follow-up.

## Data Availability

The datasets used and/or analyzed during the current study are available from the corresponding author on reasonable request.
